# Reintroducing threatened pine-associated fungal species in boreal forests

**DOI:** 10.1007/s00267-025-02166-6

**Published:** 2025-04-26

**Authors:** Joette Crosier, Reijo Penttilä, Otto Miettinen, Brendan Furneaux, Jorma Pennanen, Leena Hamberg

**Affiliations:** 1 Forest Health & Biodiversity Unit, Natural Resources Institute Finland (Luke), Helsinki, Finland; 2 Environmental, Food, & Biological Sciences, University of Helsinki, Helsinki, Finland; 3 Department of Biological & Environmental Science, University of Jyväskylä, Jyväskylä, Finland

**Keywords:** Red-listed, Wood-decay fungi, Conservation, Inoculation, Finland

## Abstract

Many species of wood-inhabiting fungi, particularly in the boreal forests of Nordic countries, face significant extinction risks. The historical impact of commercial forestry has led to fragmented old-growth forests, meaning that species lost from these areas may not naturally return to newly protected habitats. This study investigates the potential of inoculation as a management tool to aid the reintroduction of threatened fungal species. Specifically, we tested whether red-listed fungal species associated with dead pine wood could successfully establish in inoculated pine logs and identified factors influencing inoculation success. We cultured mycelium from five target species (*Anthoporia albobrunnea, Antrodia crassa, Antrodia infirma, Crustoderma corneum, Dichomitus squalens*) and inoculated pine logs in selected forests, monitoring log characteristics and conducting DNA analysis of the fungal community before and after inoculation. Our findings demonstrate that all species successfully established in at least some of the inoculated logs (28–60% success), with variable effects of log characteristics on fungal abundance. Additionally, the presence of certain fungi correlated with the success of the inoculated species. These results suggest that inoculation can be a promising method for aiding the recovery of threatened wood-inhabiting fungi in appropriate forest habitats. Long-term monitoring is necessary to assess fruiting success and population sustainability, while further exploration of alternative techniques could enhance the effectiveness of reintroduction efforts in forest management practices.

## Introduction

Current forestry practices are destroying vast habitats through tree clearing and management strategies that leave minimal deadwood. Unmanaged boreal forests can have 50–120 m^3^ of deadwood per hectare (Siitonen, 2001), while commercial forests contain only 5–7 m^3^ (Jonsson et al., 2016; Korhonen et al., 2021). In Finland, only 13% of forests are protected (Natural Resources Institute Finland, 2022), leaving the rest available for logging.

An estimated 4000–5000 Finnish species, including many wood-inhabiting fungi, depend on deadwood (Siitonen, 2001; Stokland et al., 2012). These fungi face local extinctions as they exhaust substrates and can only survive by colonizing new ones (Jönsson et al., 2008). Fragmentation of old-growth forests particularly threatens red-listed species (Penttilä et al., 2006; Abrego et al., 2015; Nordén et al., 2013), making consistent deadwood availability crucial for landscape-level management. Polypore species (Basidiomycete fungi with the spore-bearing surface consisting of pores or tubes) diversity is highly dependent on diverse and abundant deadwood, with many red-listed polypore species only persisting in forests with many times higher amounts of deadwood than found in managed forests (Penttilä et al., 2004; Junninen & Komonen, 2011). For pine-inhabiting fungi, deadwood quality is even more important than for fungi inhabiting other boreal tree species (Niemelä et al., 2002).

Fungal conservation is an emerging area in biodiversity management, with global fungal red-listing only becoming prominent in 2019 (Pasailiuk et al., 2022). In-situ and ex-situ fungal conservation have a shorter history compared to plants and animals (Pasailiuk et al., 2022). However, building fungal conservation capacity is critical for biodiversity and ecological preservation. To conserve rare and threatened fungi, habitat loss must be stopped, and more protected forests must be created. Forest management should actively promote deadwood accumulation, a major goal in Europe’s boreal forests (Similä & Junninen, 2012), and even supplement natural processes (Lonsdale et al., 2008). However, habitat protection alone may not suffice to conserve red-listed fungi (Pasanen et al.,^ 2014^), which include 40% of Finnish polypores (Kotiranta et al., 2019). Dispersal limitation is a challenge, as spores typically travel only a few meters, preventing fungi from colonizing fragmented landscapes (Möykkynen et al., 1997; Nordén & Larsson, 2000; Norros et al., 2012), and though some spores travel further, dispersal is driven largely by stochastic processes and can, at the landscape scale, be limiting at the tens or hundreds of meters range (Peay & Bruns, 2014). Germination requirements and competition also reduce fungal colonization (Edman et al., 2004). Reintroduction of species can be an effective complementary conservation strategy when used appropriately (IUCN/SSC, 2013). Although limited, studies suggest fungal inoculation is a viable tool for forest biodiversity restoration (Piȩtka, Grzywacz (2005); Piȩtka, Grzywacz (2006); Abrego et al., 2016), and interest in the method is growing (Nordén et al., (2020)).

Log characteristics and microclimate influence fungal growth (Rayner & Boddy, 1988). Many species have specific substrate preferences and are sensitive to wood conditions (Toljander et al., 2006). Tree diameter is a key factor; rare wood-decay fungi are often found more in large-diameter trees (Renvall, 1995; Stokland & Kauserud, 2004; Penttilä et al., 2013), but species richness is generally higher in smaller trees when compared per volume unit (Heilmann-Clausen & Christensen, 2004; Juutilainen et al., 2011), even for red-listed species, some of which occur on small branches (Martikainen et al., 2000; Korhonen et al., 2024). This highlights the need for variable deadwood sizes to support diverse species.

Decay stage of the host log also impacts the fungal community. Regarding threatened wood-decaying fungi, fruit body surveys show that there is a trend of higher occurrence for logs with intermediate or late decay levels (Bader et al. 1995, Renvall 1995, Junninen & Komonen, 2011) and in Norway spruce (*Picea abies*) logs there is generally high species and sporocarp richness at the intermediate decay stages (Bader et al. 1995, Jönsson et al. 2008). However more recent DNA sequencing has shown that, while overall fungal richness is highest in the later stages, known wood-decayers (mainly Basidiomycetes) are most abundant in earlier decay stages (Kubartova et al. 2012). Lab studies suggest that late-stage fruiting fungi may outcompete early-stage fungi (Holmer & Stenlid, 1997), meaning rare fungi could exist as mycelia in early stages but only fruit when conditions change.

In this study, we investigated the reintroduction potential of five species of locally red-listed (Kotiranta et al. 2019) wood-decay fungi (*Anthoporia albobrunnea, Antrodia crassa, A. infirma, Crustoderma corneum*, and *Dichomitus squalens*). The first four are specialists of fallen *kelo* logs. These logs are formed from decorticated pine (*Pinus sylvestris*) snags (*kelo* in Finnish) which stand vertically even for decades after death, decaying slowly and dropping all or most of their bark before finally falling down (Niemelä et al. 2002). The fifth species (*Dichomitus squalens*) prefers storm damage and forest fire areas with abundant dead conifer wood.

The aim of this project is to test the inoculation method for conserving threatened fungi associated with pine kelo trees in southern Finland. It also explores ecological factors influencing inoculation success, specifically decay stage, tree diameter, and wood quality (kelo vs. regular wood).

Building on Abrego et al. (2016), we hypothesize that these fungi can be successfully reintroduced to forests via log inoculation. Although the species in our study are typically found on more decayed logs, we hypothesize that fungi inoculated into decay stage one logs will perform better than those in decay stage two. In Abrego et al. (2016), inoculations of spruce-living polypores (mostly late-fruiting species) were most successful in stage one logs. We hypothesize that log diameter will have no impact on the initial establishment success, becoming important only later in life for our species, but we hypothesize that fungi inoculated on kelo logs will have an advantage allowing them to establish better than those inoculated on regular pine logs.

## Material and methods

### Fungal species and forest sites

For our reintroductions, we used five red-listed pine specialist basidiomycete species (*Anthoporia albobrunnea, Antrodia crassa, Antrodia infirma, Crustoderma corneum, Dichomitus squalens*). One of them (*D. squalens*) is a white-rot fungus and the rest brown-rot fungi. We used multiple strains of every species, to address genetic diversity (Table 1).

**Table 1:** The date, locality, and coordinates of the origin place where each strain was collected. Fungal strains used in the study were collected from different parts of southern or central Finland

Species	Strain ID	Date of first culture	Municipality	Coordinates (WGS84)
*Anthoporia albobrunnea*	JPC 133	September 2018	Kuhmo	63.9871, 30.3417
	JPC 99	September 2018	Kuhmo	63.9864, 30.3468
	JPC 51	September 2018	Hämeenlinna	61.2409, 25.0634
	JPC 49	October 2018	Lieksa	63.1497, 30.7024
*Antrodia crassa*	OMC 1865	October 2018	Suomussalmi	65.4654, 29.2539
	JPC 178	October 2018	Suomussalmi	65.4441, 29.6026
	JPC 171	October 2018	Suomussalmi	65.4441, 29.6026
*Antrodia infirma*	JPC 54	August 2018	Sotkamo	63.8810, 29.1182
	JPC 58	September 2018	Kuhmo	63.9904, 30.3332
	JPC 17	October 2018	Kuhmo	63.9851, 30.3428
	JPC 137	October 2018	Lieksa	63.1498, 30.7199
*Crustoderma corneum*	JPC 176	October 2018	Lieksa	63.1486, 30.6910
	JPC 130	October 2018	Suomussalmi	65.4452, 29.5991
	JPC 182	October 2018	Ikaalinen	61.9113, 23.4065
	JPC 183	October 2018	Ikaalinen	61.9118, 23.4049
*Dichomitus squalens*	JPC 154	October 2018	Ilomantsi	62.9394, 31.4287
	JPC 138	October 2018	Ilomantsi	62.9378, 31.4307
	JPC 140	October 2018	Ilomantsi	62.9995, 31.4138

Our inoculation work was done in four protected forest sites from southern and central Finland: Rokua National Park (64.55° N, 26.51° E), Nuuksio National Park (60.29° N, 24.55° E), Keurunmäki-Haavikkolehto (62.54° N, 26.91° E), and Petkeljärvi National Park (62.59° N, 31.17° E). All sites are state owned and managed by Metsähallitus, the Finnish Forest Administration. The forests were chosen not to represent high-quality old-growth forests with a very long continuity and abundance of deadwood and associated fungal species. Rather, they are pine-dominated, protected forests which contain enough slightly decayed deadwood for the inoculation purposes and deadwood continuum in the future. In addition, we reviewed results from the fungal inventories made in previous years to confirm that the sites have no or very few findings of the inoculated species.

Before inoculations, all study logs were surveyed carefully to verify that they did not host fruit-bodies of the inoculated species. In addition, the forest site and its immediate surroundings (~100 meters away from each inoculated log) were surveyed with the intention to select areas without or with a very low abundance of the target species. Surveys revealed presence of target species in low quantities in some of the sites; *Antrodia crassa* was found once (Petkeljärvi National Park), *Antrodia infirma* was found twice (Rokua National Park), *Crustoderma corneum* was found twice (Petkeljärvi National Park and Keurunmäki-Haavikkolehto), and *Dichomitus squalens* was found once (Nuuksio National Park).

### Cultivations in the laboratory

After strains were gathered from the field, pure cultures were produced by adding internal pieces of the fruit body or spores released form fruit-bodies to Petri dishes of 2% malt extract agar (MEA) and transferring clean hyphae to a new similar MEA dish. Mycelia on agar plates were allowed to grow out to have enough starting biomass for use, between two weeks and one month for these species. DNA was extracted from the hyphae of the pure cultures, according to the manufacturer’s instructions (FastPrep), and Sanger-sequenced to verify species identification (See Appendix 1 for more details).

Each fungal strain was cultivated on five MEA Petri dishes. The dishes were then used to inoculate first stage spawn, as this is standard practice in mushroom spawn production for quick and reliable dowel colonization. In this case, we used rye grain that had been soaked to the appropriate moisture content and then sterilized at 121 °C and 15 PSI for 3 h and 20 min. After the grain was fully colonized by mycelium, it was used to inoculate the final spawn. In this case, we used 1 × 5 cm round dowels made of pine wood. The dowels were soaked to reach a suitable moisture content (~60%), and then sterilized at 121 °C and 15 PSI for 2 h and 40 min. Myceliated grain spawn was added to the prepared dowels at a rate of around 5%. Mycelium was then allowed to grow on the dowels until fully colonized, at which time they were packed into smaller bags to carry to the forest sites. Both stages of spawn – grain and dowels – grew for 1–3 weeks each.

### Inoculations and other measurements in the field

For every species, we inoculated the fungi into kelo logs, the natural preferred substrate of the fungi, and normal pine logs with more or less intact bark (Table 2). It was not possible to inoculate *Antrodia infirma* to any logs in Petkeljärvi National Park, because according to prior information the species was recently found in high abundance (~10 occurrences) in that forest. To compensate for less *A. infirma* inoculations there, extra logs in Keurunmäki-Haavikkolehto were inoculated with *A. infirma*. None of the logs that were inoculated were found to contain the target species, except for one log which after DNA analysis showed a small presence of *A. infirma* before the inoculation.

**Table 2:** Number of kelo and normal pine logs inoculated per species in each site

	Site			
	Rokua National Park	Nuuksio National Park	Keurunmäki-Haavikkolehto	Petkeljärvi National Park
Species	Kelo/Non-kelo	Kelo/Non-kelo	Kelo/Non-kelo	Kelo/Non-kelo
*Anthoporia albobrunnea*	4/4	4/4	4/4	4/4
*Antrodia crassa*	4/4	4/4	4/4	4/4
*Antrodia infirma*	4/4	4/4	4/8	0/0
*Crustoderma corneum*	4/4	4/4	4/4	3/4
*Dichomitus squalens*	4/4	4/4	4/4	4/4
Control	4/4	4/4	4/4	5/4

**Fig. 1:**
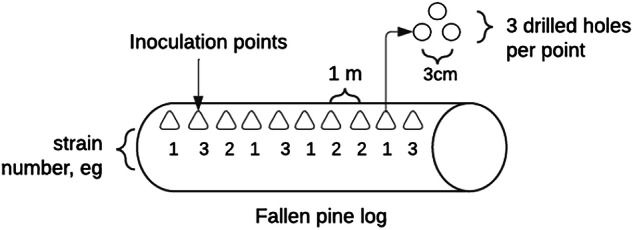
Layout for inoculations into pine logs. Altogether 60 dowels (2 per hole) including one of the fungal species were put inside each log

Inoculations were carried out between August and October of 2020. Each log had ten inoculation points, each of which consisted of three holes in a triangle shape, and inoculation points spaced one meter apart (Fig. 1). Inoculation was carried out by first drilling a hole, inserting two dowels per hole to inoculate 10 cm deep, and hammering them into the hole with a mallet, and covering the point with garden wax (Neko Ab, Finland). We added one species per log, and every strain of that species was inoculated into each log, in randomly assigned alternating patterns. We also created control logs, in which we drilled the same pattern of inoculation points, but added only uninoculated dowels. Sawdust drilled out from each log was collected and combined to one sample and transported to subsequent laboratory analysis.

At the time of inoculations, we collected data on the characteristics of each log: whether the log was fallen just above the base of a tree or uprooted, diameter in cm (i.e., 1.3 m from the base of the tree), decay stage, log contact with the ground (%), and intact bark cover (%). Decay stage, the level of decomposition in a log, was determined using a five-level classification system (Renvall, 1995) in which decay stage 1 is the least decayed and stage 5 is the most decayed.

One year after inoculations were carried out, we returned to the field sites to take samples from the inoculated logs. Samples were taken similarly to the previous ones. We drilled out sawdust at two holes per inoculation point, where each new drill hole was situated in line approximately 1.5 cm away from the dowels, one in between the single top hole and the right-hand bottom hole, and one in between the two bottom drill holes. Again, all sawdust samples per one log were combined and sent for subsequent laboratory analysis.

### Laboratory processing

In the laboratory, sawdust samples from both the time of inoculation and one year later were prepared for DNA analyses by first applying parafilm to the tubes and freeze drying (62–92 h at 0.57 mbar vacuum and shelf temperature of 15 °C). Then samples were placed into metal grinding jars (washed with DNA/RNA decontamination solution) with heat sterilized metal balls (a mix of 2 and 10 mm). Using a Mixer Mill MM 400 (Retsch) homogenizer, samples were pulverized at a frequency of 30 s^−1^ for five minutes at a time, for a total time between 5 and 30 min, as needed to reach fine enough consistency. Pulverized samples were then added to the final tubes (2 ml sawdust each) to be sent for DNA extraction and analysis, which was carried out at the Canadian Centre for DNA Barcoding (extraction and amplification) and the Advanced Analysis Centre (next generation sequencing) at the University of Guelph, Canada. We used the internal transcribed spacer 2 (ITS2) region with spiking approach for DNA analysis and quantitative estimates (see Ovaskainen & Abrego 2020). Additional details can be found in Appendix 1. DNA sequences can be accessed at NCBI Sequence Read Archive, Project: PRJNA1096717.

### Bioinformatics

Bioinformatics was performed using a development version of the OptimOTU pipeline, as described briefly in Saine et al. (2024), with some additional modifications. In overview, the steps of the pipeline included trimming, quality filtering, denoising, and merging of raw paired end reads to form amplicon sequence variants (ASVs); additional trimming and removal of positive control sequences and chimeric sequences from the ASVs; taxonomic identification using ProtaxFungi (Abarenkov et al. 2018); taxonomically-informed clustering to form OTUs; removal on non-fungal OTUs; and assignment to ecological guilds using the FUNGuild database (Nguyen et al. 2016).

When available, Sanger sequences of the strains used in the study, as well as additional sequences of the target species from the Natural Resource Institute Finland’s internal culture collection, were incorporated into the ProtaxFungi reference database prior to taxonomic identification in order to improve identification of these species. For several of the target species, the ProtaxFungi database used a synonym for the species (Appendix 1). The ProtaxFungi version of the classification was used for additions to the reference database, but results for these species are reported using the currently preferred names, and synonyms were identified using MycoBank (Robert et al. 2013). Additional details on the bioinformatics process are in Appendix 1.

### Data handling and statistical analyses

The term ‘success’ was used to describe the occurrence and relative abundance of OTUs of the inoculated fungal species, indicating the ability for the mycelium to grow at least 1.5 cm away from the inoculation points (determined by presence of the inoculated species after sequencing in a combined and homogenized sample along the log).

First, we compared presence of target species before and after inoculation, by counting the number of logs which did not contain any reads of the target species before inoculation and counting how many of those same logs contained reads of the target species one year after inoculation. Then we calculated what percentage of logs showed any degree of colonization.

Since some log characteristics correlated strongly with each other (Appendix 3), only uncorrelated explanatory variables (*r* < 0.5) were included in statistical models. Before running models for log characteristic-based success, the OTU raw data was translated into quantitative estimates using the values from the spiking approach (where a known quantity of positive control was included to account for sequencing depth, detailed in Appendix 1). These quantitative estimates are referred to as “abundance” throughout. We used generalized additive mixed models (GAMMs) with Gaussian distribution to test the effect of log characteristics on the abundance (quantitative estimates of OTUs) of the inoculated target species – each species separately – in the statistical program R version 4.2.1 with *gamm4* package (R Core Team 2022, Wood & Scheipl 2020). As explanatory variables we included (1) wood quality (a factor with two levels: 0=not kelo, 1=kelo) (2) decay stage (a factor with two levels: 1=decay stage 1, 2=decay stage 2), and (3) diameter (cm) as a smoothed term. We could not include bark cover, ground contact, and method of tree falling (broken or uprooted) in the models, as they were strongly correlated with the kelo factor or other variables included in the models (Appendix 3). The forest site was included as a random factor in the models. For *A. albobrunnea*, the model could not be estimated because of the low number of successful observations. We checked all models using *gam.check* and *plot.gam* from *mgcv* package (v1.8-34; Wood 2011) to evaluate indicators of model success such as edf and k-index, and to visually inspect the residuals plots to ensure that model assumptions were fulfilled. Figures of the log characteristic models were drawn with the package *ggplot2* (Wickham 2016) using predicted values and associated standard errors from the *predict.gam* function in the *mgcv* package (v1.8-34; Wood 2011).

We separately investigated whether the fungi already present within the log had any positive or negative associations with the establishment success of our inoculated species. For this analysis, we first removed species which were likely to be contaminants, such as lichens and lichenicolous fungi. We then combined species into genera to decrease the high presence of zero values and get more accurate models. We only included genera that occurred in at least 50% of our observations and with a prevalence of at least 0.5% in the total OTU counts in our models. For the statistical analysis, we used joint species distribution models, which are multivariate hierarchical generalized linear mixed models fitted with Bayesian inference (Ovaskainen & Abrego 2020), using the *Hmsc* R package, version 3.0-13 (Tikhonov et al. 2022). For each model set -consisting of one model per genera, and of which there was one set per inoculated species- we used the abundance of each other genera (read counts) present in the log as the response variables and the occurrence of our inoculated species, recorded as absence or presence, as the explanatory variable. Total read count was included to account for sequencing depth, and the random factors were site (*n* = 4) and sample (*n* = 31 for *Anthoporia albobrunnea*, *n* = 32 for *Antrodia crassa*, *n* = 27 for *Antrodia infirma*, *n* = 31 for *Crustoderma corneum*, and *n* = 32 for *Dichomitus squalens*). A lognormal Poisson distribution was used to estimate the models. The models were estimated by using two Markov Chain Monte Carlo (MCMC) chains with 1,050,000 iterations. The first 150,000 iterations were discarded as burn-in, and the rest were thinned by 300 yielding altogether 3000 posterior samples per chain. Potential scale reduction factor (PSRF) of parameters and posterior trace plots were examined to verify the convergence of MCMC chains (Ovaskainen and Abrego 2020, pp. 75–76). Finally, variance partitioning in *Hmsc* R package, version 3.0-13 (Tikhonov et al. 2022) was used to estimate the explanatory power of different factors within the log compared to all other factors.

## Results

One year after inoculations, a portion of the total inoculated logs had a detectable presence of the target species: *Antrodia crassa* (59%), *Antrodia infirma* (57%), *D. squalens* (47%), *C. corneum* (36%), *Anthoporia albobrunnea* (29%), broken down by log characteristics in Table 3. None of the species were detected in the initial samples from the control logs, but *A. infirma* was detected in two control logs one year after “inoculation” with sterile dowels (no fungal species).

**Table 3:** The raw values of logs in which we detected our inoculated species one year after inoculation shown by log characteristics

	*Anthoporia albobrunnea*			*Antrodia crassa*			*Antrodia infirma*			*Crustoderma corneum*			*Dichomitus squalens*		
Characteristic	success	total	success %	success	total	success %	success	total	success %	success	total	success %	success	total	success %
Kelo	4	16	**25.0**	10	16	**62.5**	8	12	**66.7**	6	16	**37.5**	8	16	**50.0**
Non-kelo	5	16	**31.3**	9	16	**56.3**	8	16	**50.0**	5	15	**33.3**	6	16	**37.5**
Fallen (uprooted)	4	16	**25.0**	8	16	**50.0**	9	18	**50.0**	4	15	**26.7**	5	15	**33.3**
Fallen (broken)	5	16	**31.3**	11	16	**68.8**	7	10	**70.0**	7	16	**43.8**	10	17	**58.8**
Decay Stage 1	7	22	**31.8**	18	25	**72.0**	12	19	**63.2**	9	22	**40.9**	11	24	**45.8**
Decay Stage 2	2	10	**20.0**	1	7	**14.3**	4	9	**44.4**	2	9	**22.2**	4	8	**50.0**

Shown here is the total number of logs inoculated for each characteristic by species, and “success”, which in this case, is the number of logs which had a detectable presence of the inoculated species, regardless of quantitative estimates of abundance of that species as used in the models

**Fig. 2:**
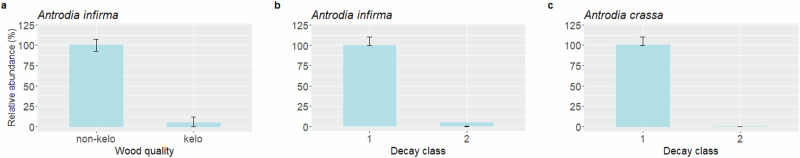
Predicted abundance (quantitative estimates of DNA) of inoculated species **a** in pine logs with different wood qualities (when the abundance in non-kelo has been set to 100), and **b, c** in pine logs with different decay states (scaled so that abundance in decay stage 1, i.e., in less decayed logs, is 100). The figure was drawn using the calculated predicted values based on the estimated models, and standard error bars are from the SE of the predicted values. Figure **a** has been calculated for decay class 2 with average log diameter, and figure **b** was calculated with non-kelo logs with average log diameter

**Fig. 3:**
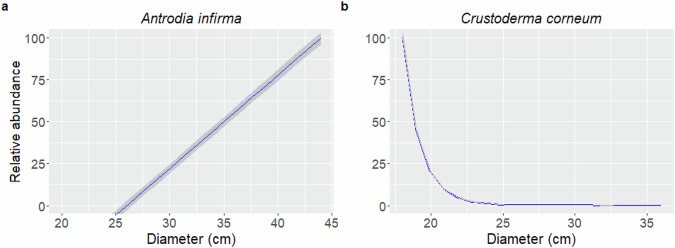
Predicted quantitative estimates of OTU abundance of **a** *Antrodia infirma* and **b** *Crustoderma corneum* relative to log diameter, with values scaled to a maximum abundance of 100. The figure was drawn using the predicted values based on the estimated model, and calculated for non-kelo logs in decay class 2. The shaded area represents the standard error of mean of the predicted values

While *Antrodia infirma* was found more often in inoculated kelo than non-kelo logs (67% and 50% respectively), growth in the kelo wood was significantly worse for this species (*p* = 0.033) with an 82% lower abundance compared to non-kelo wood (Table 4, Fig. 2). Inoculation in decay stage 2 had a significantly negative impact on *A. infirma* and *Antrodia crassa* compared to decay stage 1 (*p* = 0.033 and 0.015, respectively). Larger log diameter was significantly positively associated with stronger establishment of *A. infirma* and negatively associated with the establishment of *Crustoderma corneum* (Table 4, Fig. 3). Other significant results in terms of these variables were not found.

**Table 4:** The effects of log characteristics on quantitative estimates of relative abundance of inoculated species

Species	N	Intercept		Wood is Kelo		Decay stage 2		Diameter
		Coeff. ± SE	*p*	Coeff. ± SE	*p*	Coeff. ± SE	*p*	*p*
*Antrodia crassa*	32	**−5.468** ± **1.635**	**0.002**	0.723 ± 2.243	0.750	**−6.463** ± **2.505**	**0.015**	0.553
*Antrodia infirma*	28	**5.108** ± **2.439**	**0.047**	**−3.344** ± **−2.270**	**0.033**	**−4.329** ± **−2.270**	**0.033**	**0.010**
*Crustoderma corneum*	31	**−9.180** ± **2.354**	**0.001**	0.634 ± 1.660	0.705	−1.439 ± 1.936	0.463	**0.003**
*Dichomitus squalens*	32	**−7.928** ± **2.362**	**0.002**	1.070 ± 2.235	0.636	0.463 ± 2.753	0.868	0.131

Model coefficients (Coeff.) with standard errors of mean (SE) are presented, except for diameter, for which (as a smoothed term) only a *p* – value is presented. Statistically significant results (*p* < 0.05) are in bold

Our target species had largely variable associations with other fungi in the logs (Table 5). None of the fungal genera present had a significant interaction with the establishment of *Antrodia crassa*. *Mucor* spp. had a weakly significant (*P* = 0.91) negative association (posterior probability) with *Antrodia infirma*, and *Tympanis* spp. had a strongly significant (*P* = 0.99) positive association with the establishment of *Anthoporia albobrunnea*. Three fungal genera had a significant positive association with *Crustoderma corneum*, while *Dichomitus squalens* had six positively associated genera.

**Table 5:**
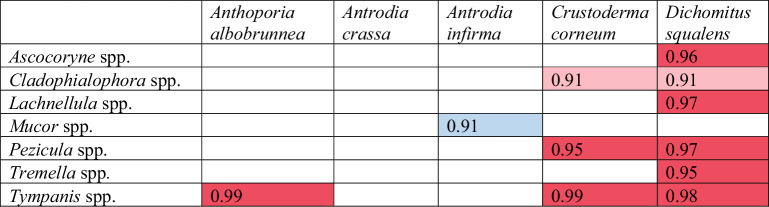
These genera were found to be associated with the success of at least one of the rare inoculated species

Red signifies a positive association and blue signifies a negative association. Dark color indicates a stronger association (with a posterior probability, *P* ≥ 0.95) and light color indicates a weaker association (0.90 ≤ *P* < 0.95). Blank squares had no significant association. A complete list of all genera analyzed is in Appendix 4

**Fig. 4:**
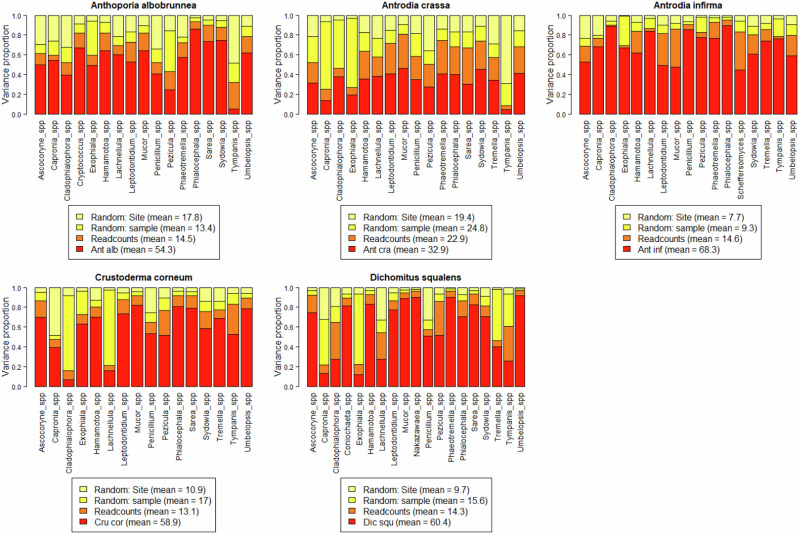
Variance partitioning of the abundances of pre-existing fungal genera based on quantitative estimates, sample (which covers the logs characteristics), site (which covers broader location effects), and total read counts. Read counts as used here was to control sequencing depth, and is also shown to have some degree of explanatory power

Variance partitioning results indicate that fungal community composition has a stronger association with the establishment success of the inoculated fungi, compared to other variables we used to characterize logs and sites. At the species level, community composition had a milder association in the case of *Antrodia crassa* (32.9%), compared to the rest in which the community accounted for more than 50% of variation Fig. 4.

## Discussion

Our study demonstrates that threatened pine-associated fungal species can successfully establish in inoculated pine logs, offering a viable management tool for their conservation. The establishment success rates of these species were comparable to the highest-performing spruce-associated species and surpassed the lowest-performing species in a recent study (Saine 2024). It is also possible that the colonization success rates we found are underestimated, in case the inoculated fungi did survive without managing to spread 1.5 cm from the inoculation point within the first year. There is not much information on how far different fungal species can grow within a natural wood substrate per time unit. A recent study looked at inoculation of *Porodaedalea pini* (not our target species) in living pine trees found an average mycelial spread of 3.4 cm per year (Edman et al., 2024), but spread in deadwood by different species could behave differently. Therefore, a false negative could be possible for exceptionally slow growing strains. Although certain wood-decay fungi found in nature seem to have strong preferences in terms of log characteristics based on fruit body surveys, our results indicate that those characteristics are not strictly necessary and may not be necessary for rare and threatened fungi to grow, if the opportunity to colonize is presented - such as through inoculations. Instead of log characteristics, factors inherent to the fungal species, such as spore productivity, germinability, or competition ability, may be more important drivers of rarity than substrate requirements.

The result that inoculation at a later decay stage has a neutral to negative impact on colonization success particularly highlights the problem with assumptions made from fruit body surveys, and reinforce the high throughput sequencing observations (Kubartova et al. 2012) which posit that many of the species can be present from early on, with rare and threatened species even able to grow in the primary colonization stage. For two species, decay stage was a significant predictor of success (*Antrodia infirma* and *A. crassa*), and as hypothesized fresher wood was better, similarly to other inoculations (Abrego et al. 2016). This contrasts with the fact that *A. infirma* is documented naturally occurring on quite rotten logs based on fruit body surveys (Kotiranta, Niemela (1996), Niemelä 2016).

Kelo wood has often been considered a prerequisite for the growth of some of our target species, but we found that the inoculated fungi could grow on both qualities (kelo and non-kelo logs). Contrary to expectations, in one instance, *Antrodia infirma*, the inoculations were even less successful on kelo wood in terms of quantity of mycelial growth based on DNA extraction and sequencing.

We also found that large diameter did not have a uniform impact across our tested species, and only effected the establishment success of *Antrodia infirma* and *Crustoderma corneum*, positively and negatively, respectively. There are mixed results based on fruit-body surveys in which some conclude that threatened fungal species had an even greater need for large diameter logs in boreal forests (Bader et al. 1995, Penttilä et al. 2013), while others found no such size preference among threatened species (Heilmann-Clausen & Christensen 2004) in a temperate forest study. One preference cannot be generalized to all species within the categorization of “threatened fungi”, so interspecific variation of the impact of a substrate characteristic is not surprising. Even considering results from previous studies, it is hard to draw comparisons between our study – which looks only at early mycelium growth – and fruit-body surveys. Most of the hypotheses for why large logs could be important for rare species are relevant to long-term persistence, such as sufficient nutrient load and slower decomposition for slow growing species, whereby the effect would matter only when the mycelium is growing in the log for a longer time and eventually, if ever, fruiting at a later stage (Heilmann-Clausen & Christensen 2004). Further, we acknowledge that unknown, correlated characteristics could contribute more to the effect on growth, rather than larger diameter itself, but further controlled study, and longer term follow up, would be needed to investigate the mechanism of action.

Conclusions we can draw from the effect of log characteristics is limited, and further study is still needed to isolate these characteristics for a more mechanistic approach. Correlating characteristics, including but not limited to those measured in our study (Appendix 3), complicate the question even in field experiments. For example, if the species are thought to be dependent on kelo wood, but as found here, kelo wood is negatively correlated with bark cover and uprooting, we cannot yet disentangle which of those characteristics are important for the fungi and to what degree. Because of the strong correlation between size and age, it is difficult to distinguish what characteristics have the most important effect on fungal growth, as density, amount of heartwood, and bark thickness all change with tree age (Stokland et al. 2012). We found that decay stage has an impact on fungal establishment, but as with age, decay stage is correlated with many other wood quality factors, including the preexisting biotic community, discussed more below. Controlling some of the log characteristics, such as using logs cut at the same time, with similar diameter and bark cover could be a useful approach to find more specific impacts of wood quality.

Biotic community composition seemed to be relatively more important than abiotic log characteristics, as indicated by the variance partitioning, which shows an overall higher impact of resident fungi than found in a similar study investigating biotic community and spruce-associated fungal inoculations (Saine et al. 2025). By assessing the model power within the HMSC framework, we can determine that target species occurrence may be more strongly explained by community interactions than environmental filtering we can measure or random processes (Ovaskainen et al. 2017) but deeper level interaction studies are suggested to parse specific community effects on inoculation success, as directional causation cannot be extrapolated from these results. While the 1.5 cm distance between annual sampling points may introduce some spatial variation, over such short distances the species pool should be very similar, especially for dominant species. In this study, we found mainly positive interspecific associations (Table 5). *Dichomitus squalens* was positively associated with a relatively high number of other fungal genera, and had no observed negative associations. Research to disentangle wood decomposition and inhabiting communities is still relatively new (van der Wal et al., (2015), Fukasawa & Matsukura 2021). The role of synergistic wood degradation and nutrient sharing in a complex setting are only beginning to be investigated, primarily in biotechnology (Rodriguez et al. 2023, Sugano et al. 2021, Lin 2022). The observed associations could result indirectly from competition between other species or from a more direct synergy, such as improved wood break-down, or other mechanisms. They could even be an artifact of similar preferences of species regarding log quality that our study did not measure. However, there are no studies investigating one-to-one species interactions between our target species and community members from our samples. Also, relatively little is known about the ecological roles of the resident fungi in our samples beyond inhabiting wood, either as saprotrophs or pathogens. Here we found that associations with some common wood inhabiting fungi exist at the local scale of inoculation sites, but to understand causal effects and interactions or a broader representative community, future experimental studies would need to be conducted. It is likely that other species of Basidiomycetes play an important role in community interactions with our target species, but unfortunately did not occur at a high enough frequency in our samples to be used in reliable models. Finally, it is crucial to note that this study did not account for bacteria in the wood, which contributes significant influence on the overall community assemblage and function (Embacher et al., (2023b), Kobayashi & Crouch 2009).

Observational studies, which make up most studies investigating rare and threatened fungi, have limitations when studying functioning of interaction mechanisms between species and environment. There has been a call for experimental studies to investigate the causal relationships between log characteristics and fungal performance (Stokland & Kauserud 2004). Our experimental study shows that observed relational trends may be more indicative of opportunistic selection or relative competitive advantage than obligatory microhabitat selection and gives information about which characteristics may be more or less important for these species.

We conclude that inoculation is a promising method for reintroducing wood-inhabiting fungi. In addition, promising results from reintroduction experiments of threatened, spruce-living wood-inhabiting fungi (Abrego et al. 2016, Saine 2024) further underline the viability of this approach in fungal conservation. However, we emphasize that long-term monitoring is essential to assess the persistence and spread of inoculated species over time. Initial establishment, as measured in this study, is just the first step. For fungal reintroduction programs, it is considerably more important whether the fungi persist and spread in the logs long enough to reach maturation and fruiting stages. For longer-term growth and fruiting, both abiotic and biotic log characteristics may have a greater impact than on initial establishment.

## Supplementary information

Appendices

## Data Availability

Sequence data of all collected samples used in this study have been deposited at NCBI Sequence Read Archive under the project code PRJNA1096717.

## References

[CR1] Abarenkov K, Somervuo P, Nilsson RH, Kirk PM, Huotari T, Abrego N, Ovaskainen O (2018) Protax-fungi: a web-based tool for probabilistic taxonomic placement of fungal internal transcribed spacer sequences. New Phytol 220:517–525 30035303 10.1111/nph.15301

[CR2] Abrego N, Bässler C, Christensen M, Heilmann-Clausen J (2015) Implications of reserve size and forest connectivity for the conservation of wood-inhabiting fungi in Europe. Biol Conserv 191:469–477

[CR3] Abrego N, Oivanen P, Viner I, Nordén J, Penttilä R, Dahlberg A, Heilmann-Clausen J, Somervuo P, Ovaskainen O, Schigel D (2016) Reintroduction of threatened fungal species via inoculation. Biol Conserv 203:120–124

[CR4] Bader P, Jansson S, Jonsson BG (1995) Wood-inhabiting fungi and substratum decline in selectively logged boreal spruce forests. Biol Conserv 72(3):355–362. 10.1016/0006-3207(94)00029-P

[CR5] Edman M, Kruys N, Jonsson BG (2004) Local dispersal sources strongly affect colonization patterns of wood-decaying fungi on spruce logs. Ecol Appl 14(3):893–901. 10.1890/03-5103

[CR6] Edman M, Eriksson AM, Carlsson F, Rydkvist T (2024) Veteranising Scots pine trees by initiating tree hollowing: Inoculation with the fungal keystone species Porodaedalia pini. Fungal Ecol 72:101375

[CR7] Embacher J, Zeilinger S, Kirchmair M, Rodriguez-R LM, Neuhauser S (2023b) Wood decay fungi and their bacterial interaction partners in the built environment – A systematic review on fungal bacteria interactions in dead wood and timber. Fungal Biol Rev 45:100305. 10.1016/j.fbr.2022.100305

[CR8] Fukasawa Y, Matsukura K (2021) Decay stages of wood and associated fungal communities characterise diversity–decomposition relationships. Sci Rep. 11(1):8972. 10.1038/s41598-021-88580-2 33903719 10.1038/s41598-021-88580-2 PMC8076174

[CR9] Heilmann-Clausen J, Christensen M (2004) Does size matter? On the importance of various deadwood fractions for fungal diversity in Danish beech forests. Forest Ecol Manag 201(1):105–117. 10.1016/S0378-1127(04)00519-5

[CR10] Holmer, L, Stenlid, J (1997) Competitive hierarchies of wood decomposing basidiomycetes in artificial systems based on variable inoculum sizes. Oikos, 79. 10.2307/3546092

[CR11] IUCN/SSC (2013) Guidelines for reintroductions and other conservation translocations. Version 1.0. (IUCN Species Survival Commission, 2013)

[CR12] Jonsson BG, Ekström M, Esseen P-A, Grafström A, Ståhl G, Westerlund B (2016) Deadwood availability in managed Swedish forests – Policy outcomes and implications for biodiversity. Forest Ecol Manag 376:174–182. 10.1016/j.foreco.2016.06.017

[CR13] Jönsson MT, Edman M, Jonsson BG (2008) Colonization and extinction patterns of wood-decaying fungi in a boreal old-growth Picea abies forest. J Ecol 96(5):1065–1075. 10.1111/j.1365-2745.2008.01411.x

[CR14] Junninen K, Komonen A (2011) Conservation ecology of boreal polypores: a review. Biol Conserv 144:11–20

[CR15] Juutilainen K, Halme P, Kotiranta H, Mönkkönen M (2011) Size matters in studies of dead wood and wood inhabiting fungi. Fungal Ecol 45(5):342–349. 10.1016/j.funeco.2011.05.004

[CR16] Kobayashi DY, Crouch JA (2009) Bacterial/fungal interactions: from pathogens to mutualistic endosymbionts. Annual Rev Phytopathol 47(1):63–82. 10.1146/annurev-phyto-080508-081729 19400650 10.1146/annurev-phyto-080508-081729

[CR17] Korhonen A, Siitonen J, Hamberg L (2024) Fungal and beetle diversity in deciduous fine woody debris in spruce-dominated forests in relation to substrate quantity and quality. Biodivers Conserv 33(14):4121–4137

[CR18] Korhonen K, Ahola A, Heikkinen J, Henttonen H, Hotanen JP, Ihalainen A, Melin M, Pitkänen J, Räty M, Sirviö M, Strandström M (2021). Forests of Finland 2014–2018 and their development 1921–2018. Silva Fennica, 55(5). 10.14214/sf.10662

[CR19] Kotiranta H Junninen K, Halme P, Kytövuori I, von Bonsdorff T, Niskanen T, Liimatainen K (2019) Aphyllophoroid fungi. In: Hyvärinen, E., Juslén, A., Kemppainen, E., Uddström, A., Liukko, U.-M. (Eds.), The 2019 Red List of Finnish Species pp. 234–247. Ministry of the Environment & Finnish Environment Institute

[CR20] Kotiranta H, Niemela T (1996) Uhanalaiset käävat Suomessa. Threatened polypores in Finland. 2nd ed. Suomen ymparistökeskus & Edita, Helsinki

[CR21] Kubartová A, Ottosson E, Dahlberg A, Stenlid J (2012) Patterns of fungal communities among and within decaying logs, revealed by 454 sequencing. Mol Ecol 21(18):4514–4532. 10.1111/j.1365-294X.2012.05723.x 22882383 10.1111/j.1365-294X.2012.05723.x

[CR22] Lin L (2022) Bottom-up synthetic ecology study of microbial consortia to enhance lignocellulose bioconversion. Biotechnol Biofuels Bioprod 15(1):14. 10.1186/s13068-022-02113-1 35418100 10.1186/s13068-022-02113-1 PMC8822760

[CR23] Lonsdale D, Pautasso M, Holdenrieder O (2008) Wood-decaying fungi in the forest: Conservation needs and management options. Eur J For Res 127(1):1–22. 10.1007/s10342-007-0182-6

[CR24] Martikainen P, Penttilä R, Kotiranta H, Miettinen O (2000) New records of *Funalia trogii*, *Perenniporia tenuis* and *Polyporus pseudobetulinus* from Finland, with notes on their habitat requirements and conservation implications. Karstenia 40(1–2):79–92. 10.29203/ka.2000.356

[CR25] Möykkynen T, von Weissenberg K, Pappinen A (1997) Estimation of dispersal gradients of S- and P-type basidiospores of Heterobasidion annosum. Eur J Pat 27:291–300

[CR26] Natural Resources Institute Finland (2022) Statistics on Forest Protection 1.1.2022. https://www.luke.fi/en/statistics/forest-protection/forest-protection-112022 (Visited on the 6^th^ of March 2024)

[CR27] Nguyen NH, Song Z, Bates ST, Branco S, Tedersoo L, Menke J, Schilling JS, Kennedy PG (2016) FUNGuild: an open annotation tool for parsing fungal community datasets by ecological guild. Fungal ecol 20:241–248

[CR28] Niemelä T, Wallenius T, Kotiranta H (2002) The kelo tree, a vanishing substrate of specified wood-inhabiting fungi. Pol Botanical J 47:91–101

[CR29] Niemelä, T (2016) Suomen Käävät. Finnish Museum of Natural History LUOMUS : Viherympäristöliitto : Suomen Puunhoidon Yjdistys

[CR30] Nordén B, Larsson KH (2000) Basidiospore dispersal in the old-growth forest fungus *Phlebia centrifuga* (Basidiomycetes). Nordic J Bot 20(2):215–219. 10.1111/j.1756-1051.2000.tb01572.x

[CR31] Nordén J, Penttilä R, Siitonen J, Tomppo E, Ovaskainen O (2013) Specialist species of wood-inhabiting fungi struggle while generalists thrive in fragmented boreal forests. J Ecol 101:701–712

[CR32] Nordén J, Abrego N, Boddy L, Bässler B, Dahlberg A, Halme P, Hällfors M, Maurice S, Menkis A, Miettinen O, Mäkipää R, Ovaskainen O, Penttilä R, Saine S, Snäll T, Junninen K (2020) Ten principles for conservation translocations of threatened wood-inhabiting fungi. Fungal Ecol 44:100919

[CR33] Norros V, Penttilä R, Suominen M, Ovaskainen O (2012) Dispersal may limit the occurrence of specialist wood decay fungi already at small spatial scales. Oikos 121:961–974

[CR34] Ovaskainen O, Tikhonov G, Norberg A, Guillaume Blanchet F, Duan L, Dunson D, Roslin T, Abrego N (2017) How to make more out of community data? A conceptual framework and its implementation as models and software. Ecol Lett 20:561–576 28317296 10.1111/ele.12757

[CR35] Ovaskainen O, Abrego N (2020) Joint species distribution modelling with applications in R. Ecology, Biodiversity and Conservation, Cambridge University Press, UK

[CR36] Pasailiuk MV, Sukhomlyn M, Gryganskyi A, Fontana N (2022) World biota conservation vs fungal conservation practice. J Fungal Biol 12(1):268–284

[CR37] Pasanen H, Junninen K, Kouki J (2014) Restoring deadwood in forests diversifies wood-decaying fungal assemblages but does not quickly benefit red-listed species. Forest Ecol Manag 312:92–100. 10.1016/j.foreco.2013.10.018

[CR38] Peay KG, Bruns TD (2014) Spore dispersal of basidiomycete fungi at the landscape scale is driven by stochastic and deterministic processes and generates variability in plant–fungal interactions. New Phytol 204(1):180–191 24975121 10.1111/nph.12906

[CR39] Penttilä R, Siitonen J, Kuusinen M (2004) Polypore diversity in managed and old-growth boreal Picea abies forests in southern Finland. Biol Conserv 117:271–283

[CR40] Penttilä R, Junninen K, Punttila P, Siitonen J (2013) Effects of forest restoration by fire on polypores depend strongly on time since disturbance – a case study from Finland based on a 23-year monitoring period. Forest Ecol Manag 310:508–516

[CR41] Penttilä R, Lindgren M, Miettinen O, Rita H, Hanski I (2006) Consequences of forest fragmentation for polyporous fungi at two spatial scales. Oikos 114:225–240

[CR42] Piętka J, Grzywacz A (2006) Attempts at active protection of Inonotus obliquus by inoculating birches with its mycelium. Acta Mycol 41(2):8

[CR43] Piȩtka J, Grzywacz A (2005) In situ inoculation of larch with the threatened wood-decay fungus Fomitopsis officinalis (Basidiomycota) - experimental studies. Polish Bot J 50:225–231

[CR44] R Core Team (2022) R: A language and environment for statistical computing. R Foundation for Statistical Computing, Vienna, Austria. https://www.R-project.org/

[CR45] Rayner AD, Boddy L (1988). Fungal decomposition of wood. Its biology and ecology. John Wiley & Sons Ltd

[CR46] Renvall P (1995) Community structure and dynamics of wood-rotting Basidiomycetes on decomposing conifer trunks in northern Finland. Karstenia 35:1–51

[CR47] Robert V, Vu D, Amor ABH, Van De Wiele N, Brouwer C, Jabas B, Szoke S, Dridi A, Triki M, Daoud SB, Chouchen O (2013) MycoBank gearing up for new horizons. IMA fungus 4:371–379 24563843 10.5598/imafungus.2013.04.02.16 PMC3905949

[CR48] Rodriguez A, Hirakawa MP, Geiselman GM, Tran-Gyamfi MB, Light YK, George A, Sale KL (2023) Prospects for utilizing microbial consortia for lignin conversion. Front Chem Eng 5:1086881. 10.3389/fceng.2023.1086881

[CR49] Saine S, Penttilä R, Furneaux B, Monkhouse N, Zakharov EV, Ovaskainen O, Abrego N (2024) Natural deadwood hosts more diverse pioneering wood-inhabiting fungal communities than restored deadwood. Restor Ecol 32:e14056. 10.1111/rec.14056

[CR50] Saine S, Penttilä R, Fukami T, Furneaux B, Hytönen T, Miettinen O, Monkhouse N, Mäkipää R, Pennanen J, Zakharov EV, Ovaskainen O, Abrego N (2025) Idiosyncratic responses to biotic and environmental filters in wood-inhabiting fungal communities. Ecology 106(2):e70013. 10.1002/ecy.70013 39935359 10.1002/ecy.70013 PMC11815356

[CR51] Saine S (2024) Community assembly of wood-inhabiting fungi : disentangling the role of ecological processes by experimental approaches (dissertation). Dissertationes Universitatis Helsingiensis

[CR52] Siitonen J (2001) Forest management, coarse woody debris and saproxylic organisms: Fennoscandian boreal forests as an example. Ecol Bull 49:11–41

[CR53] Similä, M, Junninen, K (Eds.). (2012) Ecological restoration and management of boreal forests—best practices from Finland. Metsähallitus, Natural Heritage Services

[CR54] Stokland J, Kauserud H (2004) Phellinus nigrolimitatus—A wood-decomposing fungus highly influenced by forestry. Forest Ecol Manag 187(2–3):333–343. 10.1016/j.foreco.2003.07.004

[CR55] Stokland JN, Jonsson BG, Siitonen J (2012) Biodiversity in deadwood. Cambridge University Press, 302–331

[CR56] Sugano J, Maina N, Wallenius J, Hildén K (2021) Enhanced Lignocellulolytic Enzyme Activities on Hardwood and Softwood during Interspecific Interactions of White- and Brown-Rot Fungi. J Fungi 7(4):265. 10.3390/jof7040265 10.3390/jof7040265 PMC8065597 33807430

[CR57] Tikhonov G, Opedal ØH, Abrego N, Lehikoinen A, de Jonge MM, Oksanen J, Ovaskainen O (2022) Hmsc 3.0: Getting started with Hmsc: high-dimensional multivariate models

[CR58] Toljander YK, Lindahl BD, Holmer L, Högberg NOS (2006) Environmental fluctuations facilitate species co-existence and increase decomposition in communities of wood decay fungi. Oecologia 148(4):625–631. 10.1007/s00442-006-0406-3 16538482 10.1007/s00442-006-0406-3

[CR59] van der Wal A, Ottosson E, Boer W (2015) Neglected role of fungal community composition in explaining variation in wood decay rates. Ecology 96(1):124–133 26236897 10.1890/14-0242.1

[CR60] Wickham H (2016) ggplot2: Elegant Graphics for Data Analysis. Springer-Verlag New York. ISBN 978-3-319-24277-4, https://ggplot2.tidyverse.org

[CR61] Wood SN (2011) Fast stable restricted maximum likelihood and marginal likelihood estimation of semiparametric generalized linear models. J R Stat Soc (B) 73(1):3–36

[CR62] Wood SN, Scheipl F (2020) gamm4: Generalized additive mixed models using mgcv and lme4. R package version 0.2-6. https://CRAN.R-project.org/package=gamm4

