# Supplementary material for: Reintroducing threatened pine-associated fungal species in boreal forests
Source: Environ Manage. 2025 Apr 26;75(8):1938–49. doi: 10.1007/s00267-025-02166-6 (PMC12259776; doi:10.1007/s00267-025-02166-6)
Supplement: Supplementary file 1 — Appendices [file 267_2025_2166_MOESM1_ESM.docx]

**Appendix 1**

Species Verification

For the Sanger-sequencing, strains were grown out on MYA agar plates until there was enough mycelium for DNA extraction. Mycelium was scraped off agar and transferred to garnet bead tubes. We used FastPrep homogenizer, and then stored the resulting samples at -80 °C. For extraction we used the E.Z.N.A.® Forensic DNA Isolation Kit (WVR D3591-02). PCR amplification was performed on the ITS region, using primers primers ITS1 (TCCGTAGGTGAACCTGCGG) and ITS4 (TCCTCCGCTTATTGATATGC) (White et al. 1990). The PCR mix was 12.5 μl Dreamtaq Green PCR Master Mix (2X), to which we added 1 μl of 1:100 diluted DNA extract, 0.125 μl of each primer (25 μM) and 11.25 μl of PCR grade water. The mixture was first denatured at 95 °C for 3 minutes, and then cycled at 95 °C for 30 seconds, 55 °C for 30 seconds, and 72 °C for one minute, for a total of 35 repetitions. The final extension was for ten minutes at 72 °C. We ran the amplicons on 1% agarose gel and completed purification. Macrogen Europe performed the sequencing, using primer ITS1, and we then checked the sequences in Geneious Prime (version 2023.0.1) and blasted against data bases of UNITE and NCBI to verify species identification.

DNA extraction of sawdust samples

Samples were arranged in the 5 ml tubes in wells of 96 sample plates with associated records. The tubes were first sterilized with 70% ethanol. For lysis, the samples were spun for 2 minutes at 5000×g before adding 2–4 ml of insect lysis buffer (ILB) (in accordance with the proportion of sawdust in the sample), 1% polyvinylpyrrolidone (PVP), and 25 µl of Proteinase K (20 mg/ml) per 1 ml of buffer. After adding all components, we centrifuged the samples for 2 minutes at 2000×g and then added them to a shaker at 100 RPM. The following incubation was first at 56°C for 2 hours and then 65°C for another 2 hours. DNA extraction was performed as in Ovaskainen et al. (2020). The lysates were centrifuged for 5 minutes at 2000×g. A subsample of 100 µl was transferred via pipette to a 1 ml deep -well plate (Eppendorf Cat No) together with with 200 ul of 5M GuSCN Plant Binding buffer.

This mixture was added to a 96-well 1 µm Glass Fiber (GF) plate (PALL) for binding DNA with the membrane and centrifuged for 5 min at 5000×g. There were multiple DNA washes, in which the first two consisted centrifuging samples at 5000g for 2 minutes, first with 300 µl of 5M GuSCN buffer and then with 300 µl of Plant Protein Wash buffer. The next two washes were done with 600 µl of Wash buffer and centrifuged at 5000×g for 5 minutes. After washing, samples were incubated at 56°C for 30 minutes. To elute the DNA from the dry membrane, we used 70 µl of 10mM TrisHCL pH 8.0 and centrifuged at 5000×g for 5 minutes.

Standard CCDB Platinum Taq Master Mix (10.5 µl) was used for the PCR, and we used the extracted DNA as template for amplification. We added the sample (2 µl) to a template -free reaction - ready premade PCR 1 plate with primers ITS3-misN6 and ITS4-misN6 (Ovaskainen et al., 2020). Each sample included nine synthetic spike-in controls (at 0.001 ng/µl) and each 96-well plate included two negative controls in which no DNA template was added to the wells.

There were two runs of PCR. In the first, initial denaturation was carried out at 94°C for 2 minutes, followed by 40 cycles of 40 seconds denaturation at 94°C, 1 minute annealing at 51°C, and 1 minute extension at 72°C, and the final extension was 5 minutes at 72°C. For the second PCR, the following cycling conditions were followed: initial denaturation at 94°C for 2 minutes, followed by 20 cycles of 40 seconds denaturation at 94°C, 1 minute annealing at 60°C, and 1 minute extension at 72°C, followed by final extension at 72°C for 5 minutes. In the second, the same mastermix was used as the first, and additionally fusion primers with standard i5 and i7 Illumina indices (N701, N702, N703, N704, N705, N706, N707, N710 and S502, S503, S505, S506, S507, S508, S510, S511, S513, S515, S516, S517). PCR 1 products were diluted 1:1 and the second PCR used 2 ul of this diluted product as a template. A Biomek FXP robot was used for transfers of DNA template and indexed primers. We used precast agarose gels using a bufferless E-gel system (Invitrogen) to check the amplicons. The amplicons were pooled without normalization, purified with AMPure beads, quantified on a Qubit 2.0 fluorometer, and checked with high sensitivity on Agilent Bioanalyzer. Finally, the sequencing was done with Illumina MiSeq with PE2x300 according to the basic manufacturer’s protocol.

Bioinformatics

Demultiplexed read pairs were first trimmed to the target amplicon region by searching for the forward and reverse primers using Cutadapt 4.2 (Martin 2011). Prior to the primer search, 16 bases were cut from the 5’ end of the R2 reads, because these bases were found to be of anomalously low quality and interfered with the primer search. Matches to the appropriate primer were required at the 5’ end of both reads, while the reverse complement of the other primer was searched for at the 3’ end but not required. Primer matches were required to include at least 10 bases, and were allowed to include up to 20% mismatches. Primer sequences were retained on the reads at this stage, but any bases occurring before the 5’ primer or after the 3’ primer (if any) were removed. At this stage read pairs where either read was less than 100bp or included “N” bases were also discarded. After trimming, the reads were subjected to a second round of quality filtering using the “filterAndTrim” function in DADA2 1.18.1 (Callahan et al. 2016), discarding read pairs where R1 had more than 3 expected errors or R2 had more than 5 expected errors, or where either read had significant matches to the PhiX genome.

Sequences were then dereplicated, denoised, and merged as described in Callahan (2020) to form amplicon sequence variant (ASV) tables, separately for each of the 12 MiSeq runs included in the study. De novo chimera detection was then performed using code from the “removeBimeraDenovo” function in DADA2 for each sequencing run, but the final consensus step was performed across all sequencing runs. After removal of chimeric sequences, the resulting ASV tables were merged. Non-chimeric ASVs which differed only by end-gaps (“no-mismatch” duplicates) were combined using the “cluster_smallmem” command in VSEARCH 2.22.1 (Rognes et al 2016) with a clustering threshold of 1, with the most abundant sequence in each cluster was retained as representative. A second round of chimera checking was then performed using the “uchime-ref” command in VSEARCH, using as the reference database Sanger sequences from Unite (Abarenkov et al. 2023), as packaged for the Unite Species Hypothesis matching pipeline (Abarenkov 2022), augmented with the sequences of the SynMock synthetic spikes (Palmer et al. 2018). After the second round of chimera removal, SynMock sequences were identified using the “usearch_global” command in VSEARCH and removed. Remaining ASVs were then taxonomically identified using ProtaxFungi (Abarenkov et al., 2018) with an augmented reference database as described in the main text. Because ProtaxFungi cannot identify sequences as being non-Fungi, an additional matching step was performed for each ASV using the “usearch_global” command in VSEARCH, with a threshold of 0.8. The same version of the Unite database was used as reference as was used for the second chimera removal step above. ASVs were categorized as either “known fungi” if the best match was to a fungus, “known non-Fungi” if the best match was to a non-fungus, or “unknown kingdom” if the best match was to a sequence which was unannotated at the kingdom level, or if there was no match found. Sequences from all three categories were retained until after the clustering step below.

After taxonomic identification, ASVs were clustered using a taxonomically informed three-step process. In the first step, ASVs with the same taxonomic identifications at a chosen probability threshold (0.9, referred to as “reliable”) were joined to form cluster cores. In the second step, taxonomically unidentified ASVs were joined to the cluster cores using the “usearch_global” command. This step was performed iteratively until no new matches were found, to approximate single-linkage clustering. However, separate cluster cores were never merged. Finally, in the third step, the remaining unidentified ASVs were clustered using single linkage clustering with the “usearch_single_linkage” function of OptimOTU version 0.3.0 (<https://github.com/brendanf/optimotu/releases/tag/v0.3.0>), using distances calculated by the “calc_distmx” command in USEARCH 11.0.667 (Edgar 2010). Each of the three clustering steps was performed at ranks from phylum to species, using taxon- and rank-specific thresholds for the second and third steps. These thresholds were optimized on identified environmental sequences from the Global Spore Sampling Project (Ovaskainen 2020) using F-measure as a target, as described in Vu et al. (2022), but implemented using the OptimOTU package. The result of the clustering process at the final, species level are referred to as OTUs and used as the unit of analysis in the rest of the paper. All OTUs within phylum-level clusters which contained more known non-Fungi than known Fungi (as categorized above) were discarded.

Species names commonly used and occurring in this paper, and their synonyms used in ProtaxFungi.

| Name used in this paper | Name used in ProtaxFungi |
| --- | --- |
| *Fomitopsis rosea* (Alb. & Schwein.) P. Karst. [MB#100801] | *Rhodofomes roseus* (Alb. & Schwein.) Kotl. & Pouzar [MB#127496] |
| *Anthoporia albobrunnea* (Romell) Karasiński & Niemelä [MB#816287] | *Antrodia albobrunnea* (Romell) Ryvarden [MB#308772] |
| *Steccherinum collabens* (Fr.) Vesterh. [MB#436329] | *Junghuhnia collabens* (Fr.) Ryvarden [MB#315979] |
| *Antrodiella citrinella* Niemelä & Ryvarden [MB#107731] | *Flaviporus citrinellus* (Niemelä & Ryvarden) Ginns [MB#106139] |
| *Gloeophyllum mexicanum* (Mont.) Ryvarden [MB#110700] | *Osmoporus mexicanus* (Mont.) Y.C. Dai & S.H. He [MB#803881] |

**Appendix 2**

|  | Number of logs by characteristic | | | | | | | | |
| --- | --- | --- | --- | --- | --- | --- | --- | --- | --- |
|  | Fallen | | Decay | | Diameter (cm) | | | | |
|  | uprooted | broken | stage 1 | stage 2 | 18-23 | 24-28 | 29-33 | 34-38 | 39-45 |
| Kelo | 19 | 69 | 62 | 26 | 23 | 32 | 22 | 8 | 3 |
| Non-kelo | 75 | 21 | 67 | 29 | 33 | 37 | 15 | 7 | 4 |

|  |  |  |  |  |  |  |  |  |  |  |  |  |  |  |
| --- | --- | --- | --- | --- | --- | --- | --- | --- | --- | --- | --- | --- | --- | --- |
| Ground Contact | | | | | | | | | | | | | | |
| % contact | 0 | 5 | 10 | 15 | 20 | 30 | 40 | 50 | 60 | 70 | 80 | 90 | 100 |  |
| Kelo | 8 | 4 | 17 | 1 | 11 | 6 | 9 | 8 | 4 | 10 | 3 | 4 | 3 |  |
| Non-kelo | 14 | 11 | 22 | 0 | 12 | 10 | 3 | 6 | 5 | 5 | 4 | 1 | 3 |  |

| Bark Cover | | | | | | | | | | | | | | |
| --- | --- | --- | --- | --- | --- | --- | --- | --- | --- | --- | --- | --- | --- | --- |
| % cover | 0 | 5 | 10 | 15 | 20 | 25 | 30 | 40 | 50 | 60 | 70 | 80 | 95 | 100 |
| Kelo | 53 | 18 | 9 | 1 | 6 | 0 | 1 | 0 | 0 | 0 | 0 | 0 | 0 | 0 |
| Non-kelo | 9 | 3 | 18 | 1 | 21 | 1 | 13 | 10 | 6 | 9 | 1 | 2 | 2 | 0 |

**Appendix 3**

Correlation strength and type between abiotic log characteristics per each target species. Highly correlated (*r* ≥ 0.5) characteristics are in bold.

*Antrodia infirma*

|  | Kelo | Bark | Ground | Fallen | Diameter | Decay |
| --- | --- | --- | --- | --- | --- | --- |
| Kelo | 1 | Polyserial | Polyserial | Polychoric | Polyserial | Polychoric |
| Bark | **-0.71** | 1 | Pearson | Polyserial | Pearson | Polyserial |
| Ground | 0.20 | 0.29 | 1 | Polyserial | Pearson | Polyserial |
| Fallen | **-0.84** | **0.60** | -0.15 | 1 | Polyserial | Polychoric |
| Diameter | 0.20 | -0.13 | -0.15 | 0.24 | 1 | Polyserial |
| Decay | 0.25 | 0.21 | 0.47 | 0.04 | 0.00 | 1 |

*Antrodia crassa*

|  | Kelo | Bark | Ground | Fallen | Diameter | Decay |
| --- | --- | --- | --- | --- | --- | --- |
| Kelo | 1 | Polyserial | Polyserial | Polychoric | Polyserial | Polychoric |
| Bark | **-0.82** | 1 | Pearson | Polyserial | Pearson | Polyserial |
| Ground | 0.09 | 0.13 | 1 | Polyserial | Pearson | Polyserial |
| Fallen | **-0.75** | **0.55** | -0.31 | 1 | Polyserial | Polychoric |
| Diameter | 0.47 | -0.31 | -0.20 | 0.20 | 1 | Polyserial |
| Decay | -0.12 | -0.35 | -0.19 | 0.14 | -0.12 | 1 |

*Crustoderma corneum*

|  | Kelo | Bark | Ground | Fallen | Diameter | Decay |
| --- | --- | --- | --- | --- | --- | --- |
| Kelo | 1 | Polyserial | Polyserial | Polychoric | Polyserial | Polychoric |
| Bark | **-0.85** | 1 | Pearson | Polyserial | Pearson | Polyserial |
| Ground | -0.05 | 0.19 | 1 | Polyserial | Pearson | Polyserial |
| Fallen | **-0.61** | 0.21 | 0.03 | 1 | Polyserial | Polychoric |
| Diameter | 0.20 | -0.16 | -0.18 | 0.12 | 1 | Polyserial |
| Decay | -0.32 | -0.02 | 0.05 | **0.78** | 0.07 | 1 |

*Dichomitus squalens*

|  | Kelo | Bark | Ground | Fallen | Diameter | Decay |
| --- | --- | --- | --- | --- | --- | --- |
| Kelo | 1 | Polyserial | Polyserial | Polychoric | Polyserial | Polychoric |
| Bark | **-0.76** | 1 | Pearson | Polyserial | Pearson | Polyserial |
| Ground | 0.22 | 0.01 | 1 | Polyserial | Pearson | Polyserial |
| Fallen | **-0.64** | 0.40 | **-0.56** | 1 | Polyserial | Polychoric |
| Diameter | -0.25 | 0.01 | -0.23 | **0.62** | 1 | Polyserial |
| Decay | 0.00 | -0.05 | -0.22 | **0.53** | 0.47 | 1 |

**Appendix 3**


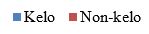


**Appendix 4**

All genera which were included in the joint species distribution models. Red signifies a positive association and blue signifies a negative association. Dark color indicates a stronger association (with a posterior probability, *P* ≥ 0.95) and light color indicates a weaker association (0.90 ≤ *P* < 0.95). If the genera did not occur in any of the logs inoculated with a given target species, this is indicated in the table with a “-“. If the box is left blank, there was no significant effect.

|  | *A. albobrunnea* | *A. crassa* | *A. infirma* | *C. corneum* | *D. squalens* |
| --- | --- | --- | --- | --- | --- |
| *Ascocoryne* spp*.* |  |  |  |  | 96 |
| *Capronia* spp. |  |  |  |  |  |
| *Cladophialophora* spp. |  |  |  | 91 | 91 |
| *Coniochaeta* spp. |  |  | - | - |  |
| *Cryptococcus* spp. |  | - | - | - | - |
| *Exophiala* spp. |  |  |  |  |  |
| *Hamamotoa* spp. |  |  |  |  |  |
| *Lachnellula* spp. |  |  |  |  | 97 |
| *Leptodontidium* spp. |  |  |  |  |  |
| *Mucor* spp. |  |  | 91 |  |  |
| *Nakazawaea* spp. | - | - | - | - |  |
| *Penicillium* spp. |  |  |  |  |  |
| *Pezicula* spp. |  |  |  | 95 | 97 |
| *Phaeotremella* spp. |  |  |  | - |  |
| *Phialocephala* spp. |  |  |  |  |  |
| *Sarea* spp. |  |  |  |  |  |
| *Scheffersomyces* spp. | - | - |  | - | - |
| *Sydowia* spp. |  |  |  |  |  |
| *Tremella* spp. |  |  |  |  | 95 |
| *Tympanis* spp. | 99 |  |  | 99 | 98 |
| *Umbelopsis spp.* |  |  |  |  |  |
